# The effect of a rapid molecular blood test on the use of antibiotics for nosocomial sepsis: a randomized clinical trial

**DOI:** 10.1186/s40560-019-0391-3

**Published:** 2019-07-22

**Authors:** Cristhieni Rodrigues, Rinaldo Focaccia Siciliano, Helio Caiaffa Filho, Cecília Eugenia Charbel, Luciane de Carvalho Sarahyba da Silva, Martina Baiardo Redaelli, Ana Paula de Paula Rosa Passetti, Maria Renata Gomes Franco, Flávia Rossi, Rogerio Zeigler, Daniel De Backer, Rafael Alves Franco, Juliano Pinheiro de Almeida, Stéphanie Itala Rizk, Julia Tizue Fukushima, Giovanni Landoni, David Everson Uip, Ludhmila Abrahão Hajjar, Tania Mara Varejão Strabelli

**Affiliations:** 1 Infectious Diseases Control, Heart Institute, University of Sao Paulo, São Paulo, Brazil; 2 Molecular Biology Division – University of Sao Paulo, São Paulo, Brazil; 3 Department of Anesthesia and Intensive Care, IRCCS San Raffaele Scientific Institute, Milan, Italy; 4 Vita-Salute San Raffaele University of Milan, Milan, Italy; 5 Microbiology Division, University of Sao Paulo, São Paulo, Brazil; 6 Department of Intensive Care at the Erasme University, Universite Libre de Bruxelles, Bruxelles, Belgium; 7 Intensive Care Unit, Instituto do Cancer, University of Sao Paulo, São Paulo, Brazil

**Keywords:** Sepsis, Blood culture, Intensive care, Antibiotic therapy, Rapid molecular test, Nosocomial infection, Critical care, Randomized controlled trial

## Abstract

**Background:**

Appropriate use of antimicrobials is essential to improve outcomes in sepsis. The aim of this study was to determine whether the use of a rapid molecular blood test—Septi*Fast* (SF) reduces the antibiotic consumption through early de-escalation in patients with nosocomial sepsis compared with conventional blood cultures (BCs).

**Methods:**

This was a prospective, randomized, superiority, controlled trial conducted at Sao Paulo Heart Institute in the period October 2012–May 2016. Adult patients admitted to the hospital for at least 48 h with a diagnosis of nosocomial sepsis underwent microorganism identification by both SF test and BCs. Patients randomized into the intervention group received antibiotic therapy adjustment according to the results of SF. Patients randomized into the control group received standard antibiotic adjustment according to the results of BCs. The primary endpoint was antimicrobial consumption during the first 14 days after randomization.

**Results:**

A total of 200 patients were included (100 in each group). The intention to treat analysis found no significant differences in median antibiotic consumption. In the subgroup of patients with positive SF and blood cultures (19 and 25 respectively), we found a statistically significant reduction in the median antimicrobial consumption which was 1429 (1071–2000) days of therapy (DOT)/1000 patients-day in the intervention group and 1889 (1357–2563) DOT/1000 patients-day in the control group (*p* = 0.017), in the median time of antimicrobial de-escalation (8 versus 54 h—*p* < 0.001), in the duration of antimicrobial therapy (*p* = 0.039) and in anti-gram-positive antimicrobial costs (*p* = 0.002). Microorganism identification was possible in 24.5% of patients (45/184) by SF and 21.2% (39/184) by BC (*p* = 0.45).

**Conclusion:**

This randomized clinical trial showed that the use of a rapid molecular-based pathogen identification test does not reduce the median antibiotic consumption in nosocomial sepsis. However, in patients with positive microbiological tests, the use of Septi*Fast* reduced antimicrobial consumption through early de-escalation compared to conventional blood cultures. These results were driven by a reduction in the consumption of antimicrobials used for Gram-positive bacteria.

**Trial registration:**

The trial was registered at ClinicalTrials.gov (NCT 01450358) on 12th October 2011

**Electronic supplementary material:**

The online version of this article (10.1186/s40560-019-0391-3) contains supplementary material, which is available to authorized users.

## Background

Sepsis is a life-threatening condition characterized by a dysregulation of the immune host response triggered by infection, and represents the leading cause of death in intensive care units [1–3]. Early administration of antibiotics for suspected infection and simultaneous antibiotic stewardship remain an essential aspect of high-quality sepsis management [4]. De-escalation of the initial empiric antimicrobial regimen based on culture data is a critical aspect of appropriate antimicrobial use [5].

The current gold-standard for blood pathogen detection is blood culture (BC). However, this method has several limitations, such as lack of rapidity and low sensitivity in case of prior antibiotic exposure, and when fastidious microorganisms are involved. These limitations result in delayed appropriate antibiotic therapy, prolonged use of broad-spectrum antimicrobial therapy, and changes in the host microbiome, thus facilitating the development of opportunistic infections and increasing selective pressure for antibiotic-resistant pathogens [6–8]. As appropriateness and timing of empirical antimicrobial therapy is essential to improve outcomes in sepsis, a faster pathogen detection would be desirable. Several molecular techniques have already been successfully developed for direct detection of virus, bacteria, and other pathogens. The LightCycler® Septi*Fast* Test (SF) is a real-time PCR-based assay capable of rapidly detecting a wide range of bacterial and mycotic pathogens. The assay uses dual fluorescence resonance energy transfer probes targeting the species specific internal transcribed spacer (ITS) regions [9].

We, therefore, conducted a randomized clinical study to evaluate whether the use of a novel molecular strategy (Septi*Fast*) for the diagnosis of sepsis directly from blood could lead to earlier more appropriate and reduced antibiotic therapy in patients with nosocomial sepsis when compared to the usual approach of diagnosis based on automated conventional blood culture system.

## Methods

### Study design

The present study was designed as a prospective, randomized, superiority, controlled trial and was conducted in accordance with The Consolidated Standards of Reporting Trials (CONSORT) Statement, approved by the local ethics and research committee (number, 0617/2011) and registered at ClinicalTrials.gov (NCT01450358).

### Patients and randomization

All adult patients (> 18 years old) admitted to the Sao Paulo Heart Institute for at least 48 h and with a diagnosis of sepsis according to the International Sepsis Definitions Conference were assessed for eligibility [10]. Exclusion occurred with at least one of the following criteria: cardiac surgery with cardiopulmonary bypass (CPB) in the last 15 days, use of intravenous heparin (due to the substantial inhibition of internal controls in Gram-negative bacilli), palliative care, and participation in other interventional studies [8, 10]. Written informed consent was obtained from all patients by a member of the local research team. After confirming the inclusion and exclusion criteria, two sets of blood cultures (aerobic and anaerobic) were collected by venipuncture according to standardized procedures. A blood sample for the LightCycler® Septi*Fast* PCR assay (SF, Roche Diagnostics GmbH, Mannheim, Germany) was also obtained prior to the initiation of antibiotic therapy and all samples were sent to the microbiology laboratory. The randomization was performed in the laboratory using a 1:1 computer-generated list created online by a web-based program that ensured allocation concealment. The nature of the intervention precluded the blinding of the attending physicians. Outcome assessors were unaware of the assigned diagnostic strategy.

### Treatment

All patients were managed according to the Surviving Sepsis Campaign guidelines [4]. In the intervention group, blood samples were immediately processed for both SF and BC. Results of the SF were available within 6 to 12 h, and antimicrobial therapy de-escalation was immediately performed accordingly, if indicated.

In the control group, blood samples were also collected for both SF and BC, but the SF sample was frozen and stored for analysis at the end of the study. Antimicrobial therapy was managed according only to the blood culture results as soon as available. Additional file 5: Figure S1 describes the details of the study design.

The institutional protocol for the empirical antimicrobial therapy initiated in all patients is illustrated in Additional file 6: Figure S2 and was managed exclusively by physicians.

An antimicrobial stewardship programme was provided by the infectious diseases team (four physicians and pharmacists) and was unchanged during the study period. At the Heart Institute, broad-spectrum antibiotics are restricted and released by the hospital pharmacy only after receiving authorization from the physicians of the infectious disease team. The microbiology laboratory results were reported once a day for 7 days a week.

### Microbiological procedures

The SF was designed to detect the microbial DNA of 25 microorganisms in whole blood samples (Additional file 1: Table S1). The test was performed in a molecular laboratory according to the manufacturer’s instructions. Further details on the microbiological procedures are available in the Additional file 8 [11].

### Data collection, definitions, and antimicrobial intervention

The following variables were recorded for each patient: age, gender, Acute Physiology and Chronic Health Evaluation-II (APACHE II) score [12], pre-existing conditions, heart failure classification, causes of hospital admission, infection site according to Centers for Disease Control [13], Sequential Organ Failure Assessment (SOFA) score [14], prior antimicrobial exposure, multidrug-resistant agent colonization, and sepsis classification [4].

Organ dysfunction was evaluated by delta SOFA, which consisted in the SOFA value on the first day of sepsis minus the SOFA value on the fourth day of sepsis [14]. The length of hospital stay was measured from the randomization date until death or hospital discharge. The time lapse between blood sample collection and reported results was analyzed.

In this trial, empirical therapy was defined as the antibiotic administration before pathogen identification from SF or BC (Additional file 6: Figure S2). Antimicrobial therapy was considered appropriate when at least one effective drug was included in the therapeutic regimen according to the identified microorganism together with susceptibility results. When only SF was used to identify the microorganism, antimicrobial therapy was considered appropriate when it contemplated the resistant bacteria coverage. Therapy de-escalation was defined as switching to a narrower-spectrum agent or decreasing the number of antibiotics to a single agent when possible [15]. Re-escalation was defined as the restart of a broad-spectrum agent or as an increase in the number of antibiotics due a clinical worsening after de-escalation of the antibiotic therapy.

### Outcomes

The primary outcome was the antimicrobial consumption during the first 14 days after randomization, expressed as days of therapy (DOT) per 1000 patients day (PD) (DOT/1000 PD). DOT per 1000 patients day was calculated as the sum of the days on therapy for all systemic antibiotics, normalized per 1000 patients day [16]. A subgroup analysis was performed for the primary outcome including only the cases with positive microorganism identification (by SF and BC in the intervention group and by BC in the control group) since only these patients received the study intervention.

Secondary outcomes were the timing of antimicrobial de-escalation, the length of hospital stay, and mortality at 10 and 28 days. Furthermore, we evaluated the costs of the antimicrobial drugs. We also calculated the diagnostic accuracy of the tools, described as sensitivity, specificity, positive predictive value, and negative predictive value.

### Statistical analysis

To detect a decrease in the days of therapy/1000 patients from 1000 in the control group to 650 in the intervention group with a standard deviation in both groups of 840 [17, 18], using a two-sided *t* test, we estimated that a sample of 182 patients was needed to achieve an 80% power at an alpha of 0.05. Considering the probability of subject attrition, we added 10% to the sample size, yielding a final required number of 200 patients. We report intention to treat analyses for the clinical outcomes. We also compared follow-up measures and clinical outcomes in patients with positive tests according to the randomized study group assignment. Continuous variables were compared using Student’s *t* test or the Mann–Whitney *U* test where appropriate, and categorical variables were compared using Pearson’s chi-square test, Fisher’s exact test where appropriate. Survival analyses were performed with a likelihood ratio test.

The results are expressed as means and standard deviation (SD) or medians with interquartile range. We calculated unadjusted Kaplan–Meier survival curves showing 28-day probability of the primary outcome for each group with the curves compared using the log-rank test.

A two-sided *p* < 0.05 was considered significant. The statistical analysis was performed using SPSS version 18.0 (SPSS Inc., Chicago, IL, USA).

## Results

### Study population

From October 2012 to May 2016, we assessed for eligibility 499 consecutive patients and 200 fulfilled the inclusion criteria, with 100 patients being randomly assigned to the intervention group and 100 to the control group. Reasons for exclusion are shown in Additional file 7: Figure S3. Baseline patient characteristics (Additional file 2: Table S2), clinical and laboratory characteristics, as well as in empirical antimicrobial therapy (Table 1) were well balanced between groups.

**Table 1:** Clinical and laboratory characteristics and empirical antimicrobial therapy of the patients

Variable	Intervention group (*n* = 100)	Control group (*n* = 100)
Presumed infection site		
Primary bloodstream infection	38 (40.4%)	43 (47.8%)
Non-ventilator associated pneumonia	20 (21.3%)	18 (20.0%)
Ventilator-associated pneumonia	15 (16.0%)	13 (14.4%)
Skin and soft tissue	6 (6.4%)	2 (2.2%)
Urinary tract infection	6 (6.4%)	3 (3.3%)
Intra-abdominal infection	1 (1.1%)	5 (5.6%)
Endocarditis	2 (2.1%)	2 (2.2%)
Surgical site infection	0 (0%)	2 (2.2%)
Pleural empyema	2 (2.1%)	0 (0%)
Other sites	2 (2.1%)	1 (1.1%)
Unknown focus	2 (2.1%)	1 (1.1%)
Severe sepsis or septic shock	83 (88.3%)	81 (90.0%)
Renal replacement therapy during sepsis	37 (37.0%)	36 (36.0%)
SOFA score, median (IQR)	7 (4–10)	8 (5–10)
C-reactive protein (mg/dL), median (IQR)	154 (106–237)	168 (104–248)
Admission lactate (mmol/L), median (IQR)	2 (1.55–2.66)	2.2 (1.55–3)
Antimicrobial exposure on time of blood collection, *n* (%)	58 (58.0%)	51 (51.0%)
Previous multidrug resistance colonization, *n* (%)	28 (28.0%)	32 (32.0%)
Empirical antimicrobial therapy		
Glycopeptides	1 (1.1%)	0 (0%)
Fluoroquinolones or 3rd-generation cephalosporins	6 (6.4%)	4 (4.4%)
Piperacillin-tazobactan or cefepime or aminoglycosides	24 (25.5%)	25 (27.8%)
Meropenem or polymyxin or tigecycline	63 (67.0%)	61 (67.8%)
Empirical MRSA coverage (glycopeptides, linezolid, and daptomycin)	93 (94.9%)	95 (95%)
Empirical Meropenem	65 (65%)	68 (68.7%)
Antimicrobial regimen		
Monotherapy	6 (6.2%)	5 (5%)
2 antibiotics	70 (72.2%)	71 (71.0%)
3 antibiotics	16 (16.5%)	20 (20.0%)
≥ 4 antibiotics	5 (5.2%)	4 (4.0%)

*SOFA* Sequential Organ Failure Assessment, *LOS* length of stay, *MRSA* methicillin-resistant *Staphylococcus aureus*, *IQR* interquartile range

### Primary outcome

In the intention to treat analysis, considering 200 patients, the median of all antimicrobial consumption, in the intervention group was 1621 (interquartile range 1196–2388) DOT/1000 PD compared to 2000 (1440–2433) DOT/1000 PD in the control group (*p* = 0.067). When analyzing antibiotic consumption according to Gram-negative or Gram-positive coverage, there were also no differences between groups (Table 2). When analyzing patients with positive test, the intervention group used significantly less antimicrobial with gram negative and positive spectrum (Additional file 4: S4 Table).

**Table 2:** Antimicrobial consumption in DOT/1000 PD during the study

Antimicrobial consumption:	All patients			Positive test		
	Intervention group	Control group	*P* value	Intervention group	Control group	*P* value
DOT/1000 PD, median, (IQR)	(*n* = 100)	(*n* = 100)		(*n* = 19)	(*n* = 25)	
- All antimicrobial	1621 (1196–2388)	2000 (1440–2433)	0.067*	1429 (1071–2000)	1889 (1357–2563)	0.017*
- Antimicrobial for Gram-negative bacteria coverage^a^	1000 (768–1466)	1071 (786–1665)	0.248*	1071 (786–1429)	1286 (536–1857)	0.427*
- Antimicrobial for Gram-positive coverage^b^	786 (554–1000)	866 (641–1000)	0.259*	71 (71–1000)	786 (354–1000)	0.013*

*Mann–Whitney test

*IQR* interquartile range

^a^Piperacillin-tazobactam, fluoroquinolones, cephalosporins, aminoglycosides, tigecycline, trimethoprim-sulfamethoxazole, meropenem, polymyxin B/E

^b^Glycopeptides, linezolid, daptomycin, andoxacillin

Patients with microorganism identification during the study were 44 (19 in the intervention group by SF and 25 in the control group by BC). The median of antimicrobial consumption in these patients was 1429 (interquartile range 1071–2000) DOT/1000 PD in the intervention group compared to 1889(1357–2563) DOT/1000 PD in the control group (*p* = 0.01) (Table 2). When analyzing antibiotic consumption according to Gram-negative or Gram-positive coverage, only the agents against Gram-positive bacteria had a significant reduced consumption in the intervention group [71 (71–1000) vs 786 (354–1000), *p* = 0.013], while for Gram-negative agents, there was no difference between groups [1071 (786–1429) vs 1286 (536–1857), *p* = 0.4].

### Secondary outcomes

The sensitivity of SF compared to BC was 72% (95% CI 55–84%), the specificity was 87% (95% CI 80–92%), the positive predictive value was 62% (95% CI 47–76%), and the negative predictive value was 91% (95% CI 85–95%). Details on the microbiological results of all included patients are described in Table 3. In Additional file 3: Table S3, we reported the identified microorganisms by SF and BC in both allocation groups.

**Table 3:** Microbiological results of all included patients

Microorganism	Microorganisms detected			
	SF only	BC only	SF + BC	Total
Included in SF detection list				
Gram-negative				
*Enterobacter cloacae*/*aerogenes*	5	–	4	9
*Escherichia coli*	2	–	1	3
*Klebsiella pneumoniae*/*oxytoca*	4	5	7	16
*Serratia marcescens*	1	–	1	2
*Acinetobacter baumanii*	–	2	1	3
*Pseudomonas aeruginosa*	3	–	2	5
*Stenotrophomonas maltophilia*	1	–	2	3
Gram-positive				
*Staphylococcus aureus*	5	1	9	15
*Coagulase negative staphylococci*^a^	–	3	1	4
*Streptococcus* spp.^b^	1	–	–	1
*Enterococcus faecalis*	–	1	–	1
Fungi				
*Candida albicans*	–	1	–	1
*Aspergillus fumigatus*	1	–	–	1
Not included in SF detection list				
*Morganella morganii*	–	1	–	1
*Burkholderia cepacea*	–	1	–	1
*Rothia* spp.	–	1	–	1
Number of microorganisms	23	16	28	67
Number of patients	17	11	28	56

^a^A group of *Staphylococcus* species (*S*. *epidermidis*, *S*. *haemolyticus*, *S*. *hominis*, *S*. *pasteuri*, *S*. *warneri*, *S*. *cohnii*, *S*. *lugdunensis*, *S*. *capitis*, *S*. *caprae*, *S*. *saprophyticus*, and *S*. *xylosus* including *S. epidermidis*, *S*. *haemolyticus*, *S*. *hominis*, *S*. *pasteuri*, *S*. *warneri*, *S*. *cohnii*, *S*. *lugdunensis*, *S*. *capitis*, *S*. *caprae*, *S*. *saprophyticus*, and *S*. *xylosus*)

^b^A group of streptococci, including *S. pyogenes*, *S*. *agalactiae*, *S*. *mitis*

Considering only the 44 patients with identified microorganisms (19 positive SF in the intervention group and 25 positive BC in the control group), the appropriate empirical antimicrobial therapy and de-escalation were not different between groups, but the time to antimicrobial therapy adjustment (8 h [7–14] vs. 54 h [38–75], *p* < 0.001) and the mean duration of antimicrobial therapy (12 ± 5 vs. 15 ± 4, p = 0.039) were lower in the SF group compared to the BC group (Table 4).

**Table 4:** Antimicrobial management in patients during the study in patients with positive tests

Variable	Intervention group	Control group	*p* value
	Positive SF	Positive BC	
	*N* = 19	*N* = 25	
Appropriate empirical antimicrobial therapy	13 (76.5%)	18 (75.0%)	1.000**
Antimicrobial de-escalation after SF or BC results, *n* (%)	17 (89.5%)	21 (84.0%)	0.700**
Antimicrobial re-escalation ≤ 7 days by clinical worsening, *n* (%)	2 (10.5%)	7 (28.0%)	0.300**
Time to antimicrobial therapy adjustment (h), median (IQR)	8 (7–14)	54 (38–75)	< 0.001*
Duration of antimicrobial therapy (days), mean (SD)	12 ± 5	15 ± 4	0.039***

*Mann–Whitney test, **Fisher’s exact test, ***T test

Microorganism identification was possible in 24.5% (45/184) by SF and 21.2% (39/184) by BC (*p* = 0.452). Considering patients with positive tests, antimicrobial costs for Gram-positive bacteria were significantly lower in the intervention group (SF) when compared to control group (BC) [68 $ (34–514) vs. 497 $ (300–552), *p* = 0.002].

In the ITT analysis of 200 patients and in patients with positive tests, there were no differences between SF and BC groups in length of hospital stay and delta SOFA (Table 5).

**Table 5:** Clinical outcomes

Variable	All patients			Positive test		
	Intervention group	Control group	*p* value	Intervention group	Control group	*p* value
	(*n* = 100)	(*n* = 100)		(*n* = 19)	(*n* = 25)	
Post-infection LOS (days), median (IQR)	19 (9–38)	16 (9–31)	0.355**	20 (10–40)	17 (9–31)	0.317**
Delta SOFA	2 (0–3)	2 (0–3)	0.882**	2 (0–3)	2 (0–3)	0.266**
Mortality						
10 days	22 (22%)	28 (28%)	0.327*	4 (21.1%)	4 (16.0%)	0.710*
28 days	40 (40%)	47 (47%)	0.318*	7 (36.8%)	11 (44.0%)	0.632*
Hospital	55 (55%)	61 (61%)	0.390*	8 (42.1%)	16 (64.0%)	0.149*

*IQR* interquartile range, *LOS* length of stay

* Pearson Chi-Square; ** Mann–Whitney

**Fig. 1:**
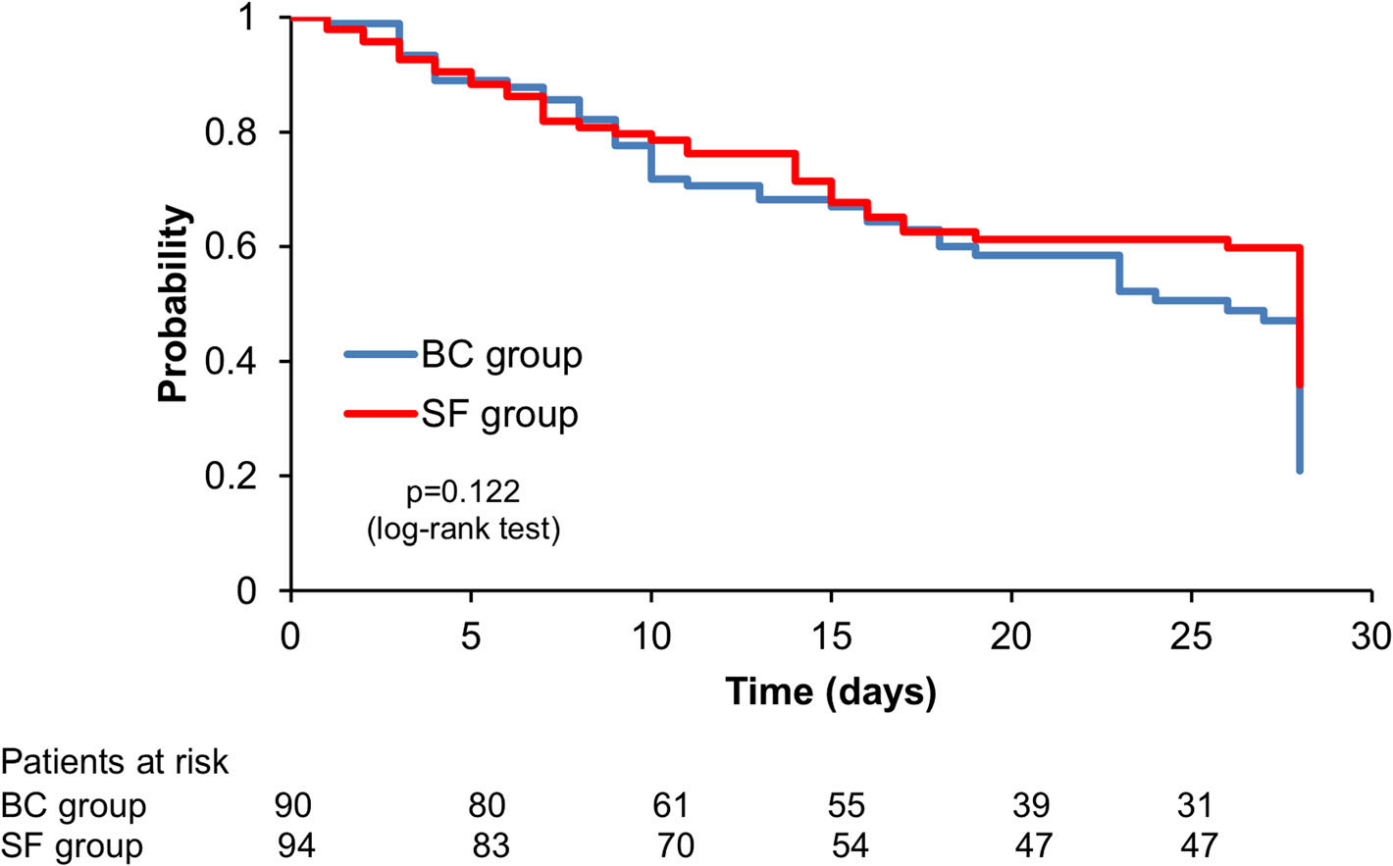
28-day Kaplan–Meier estimated survival curve in patients who underwent SF or BC tests

**Fig. 2:**
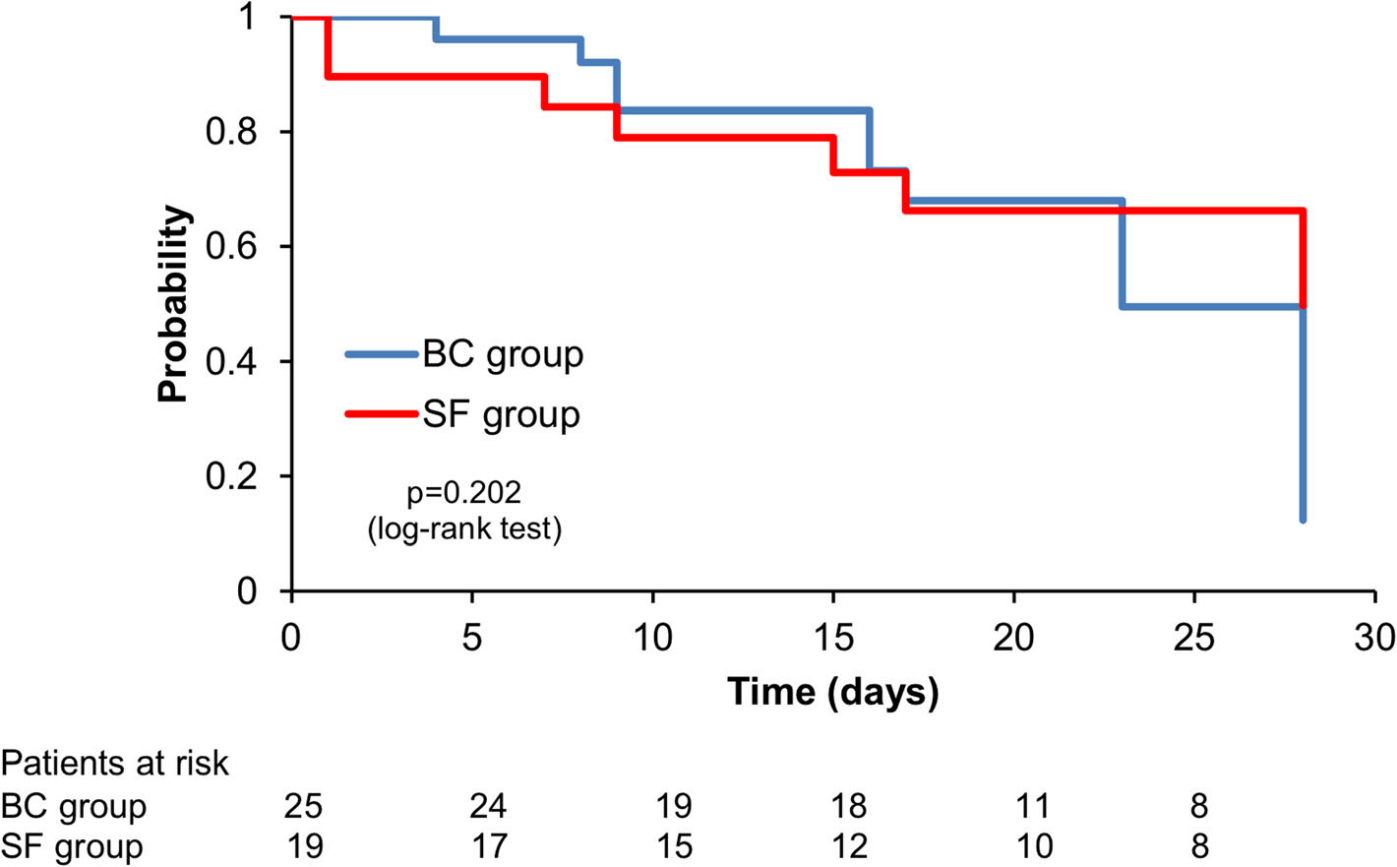
28-day Kaplan–Meier estimated survival curves in patients with positive tests (SF or BC)

The 28-day Kaplan–Meier estimated survival curves were similar between all patients in the SF group (*n* = 100) and BC group (*n* = 100) (Fig. 1) and between SF-positive group (*n* = 19) and BC-positive group (*n* = 25) (Fig. 2).

## Discussion

This randomized clinical trial showed that the use of a rapid molecular-based pathogen identification test (SF) does not reduce the median antibiotic consumption in nosocomial sepsis. However, when we analyzed only patients with positive microbiological tests, the use of Septi*Fast* reduced antimicrobial consumption through early de-escalation in patients with nosocomial sepsis compared to conventional blood cultures. These results were driven by a reduction in the consumption of antimicrobials used for Gram-positive bacteria coverage, as consequence of an earlier detection of Gram-negative bacteria by SF.

Blood cultures are the gold-standard test for the detection of microorganisms in sepsis management and provide susceptibility testing for appropriate antimicrobial therapy. However, blood cultures have some limitations, such as delay in results availability and low sensitivity (especially in patients using antibiotics), which might be associated with longer exposure to unnecessary antimicrobial therapy. Recent studies showed that BC positivity depends on multiple factors and that in nosocomial sepsis it is uncommon to found positive BC in more than 50% of cases [19].

In the last decade, new laboratory techniques for early detection and identification of microorganisms based on molecular assays were developed, and many of these techniques are commercially available [20]. The early detection of causative microorganisms in septic patients is the main target for appropriated antimicrobial therapy. This approach can be used to rapidly adjust empirical antimicrobial therapy and avoid the overuse of broad-spectrum drugs.

In the present study, the detection rates of microorganisms were similar in the SF and BC groups, thus confirming data from previous studies [21–24]. The SF group compared to the BC group showed a faster modification of empirical antimicrobial therapy, avoiding unnecessary antibiotic use and consequently reducing antibiotic consumption. Although antibiotic adjustment was performed faster using the SF test, the percentage of antibiotic de-escalation was not different between groups because the sensitivity of both methods were low and similar (SF and BC) and because the de-escalation was also based on other criteria as clinical improvement.

We showed that the use of a rapid molecular-based blood test might reduce antimicrobial consumption through early therapy de-escalation, compared to the BC-positive group, in patients with nosocomial sepsis. Within the group of patients with identified microorganism, the consumption of antibiotics with Gram-positive bacteria coverage was in fact significantly lower in the SF group compared to the BC group due to the rapid identification of Gram-negative bacteria in the blood by SF, leading to the early suspension of unnecessary antimicrobials coverage. Our main outcome was the antimicrobial consumption during the first 14 days after randomization, expressed as DOT/1000 PD. Prior studies [7, 11, 18] reported DOTS values which were usually lower than our findings because they analyzed hospital cohorts of patients with the majority of patients not receive antibiotics. In our trial, we included only patients with sepsis and therefore under antibiotic treatment. This finding explains the high value of DOTS in our study and in the manuscript of Rmawi et al. which was also performed in the intensive care unit with antibiotics prescribed to all patients [25].

The consumption of antimicrobial for Gram-negative bacteria was not different between groups, which is likely due to the use of a combined antimicrobial regimen and the absence of antimicrobial-resistance tests, avoiding an early adjustment in these patients. More recently, other multiplex PCR-based tests have become commercially available, including tests that detect resistance genes, especially in Gram-negative bacteria, such as the carbapenemase genes bla_OXA-48_, bla_VIM_, bla_IMP_, bla_NDM_, and bla_KPC_. These tests could limit the need to cover resistant bacteria when susceptibility tests are not available [26, 27]. The overall high number of days of therapy might be explained by the inclusion in our study of patients with nosocomial sepsis in a tertiary centre with high incidence of multidrug resistance bacteria colonization rates.

Our study showed that mortality rates and other clinically relevant outcomes were similar between the SF group and BC group at 10 and 28 days, suggesting that early antimicrobial de-escalating does not affect mortality and likely represents a safe strategy in septic patients, avoiding unnecessary exposure to antimicrobials, adverse events and development of further antibiotic resistances.

A previous meta-analysis, including 23 studies evaluating the efficacy and safety of antimicrobial de-escalation in septic patients, showed similar neutral results in mortality rates [14]. A recent review published by the Cochrane Library, including 221 studies (58 randomized clinical trials), demonstrates that interventions in antibiotic prescription can reduce and improve antimicrobial use and likely does not increase mortality, showing to be a safe strategy in the stewardship programme [28].

This trial was not designed to detect an impact in multidrug-resistance emergence, but early adjustment of antimicrobial therapy is a documented important strategy for resistance prevention as part of a global-appropriate antimicrobial management [29].

Our study has some limitations. It was performed in a single referral cardiology center, with a limited sample size and this may compromise the generalizability of the present findings. Moreover, the number of patients with positive microorganism identification was lower than the expected, with an actual reduction achieved in the primary outcome inferior to the 35% planned to calculate the sample size. Both these issues may explain the nonsignificant findings of our trial.

Furthermore, this rapid molecular test represents an assay with some technical difficulties limiting its use in clinical practice; however, well-trained staff can easily manage the analysis.

To our knowledge, this randomized clinical trial is the first to evaluate antimicrobial consumption using a rapid molecular test for sepsis diagnosis. Our data suggests that a rapid molecular test is a useful tool in the management of nosocomial sepsis patients to promote early appropriate antibiotic therapy and reduce the consumption of antimicrobials. The use of these new diagnostic assays might be useful to improve the outcome in septic patients and minimize unnecessary and inappropriate antimicrobial therapy, reducing the time of empirical antimicrobial use and improving healthcare quality.

## Conclusions

This randomized clinical trial showed that the use of a rapid molecular-based pathogen identification test (Septi*Fast*) does not reduce the median antibiotic consumption in nosocomial sepsis. However, in patients with positive microbiological tests, the use of Septi*Fast* reduced antimicrobial consumption through early de-escalation compared to conventional blood cultures. These results were driven by a reduction in the consumption of antimicrobial used for Gram-positive bacteria coverage. Further studies to investigate the role of molecular-based tests in improving clinical outcome are requested, as well as high sensitivity molecular based tests, in order to apply antimicrobial stewardship in sepsis.

## Additional files

Additional file 1: **Table S1.** Microorganisms detected by the LightCycler® Septi*Fast* assay. Abbreviations: CoNS: coagulase-negative *Staphylococcus*species (*S. epidermidis*, *S. haemolyticus*, *S. hominis*, *S. pasteuri*, *S. warneri*, *S. cohnii*, *S. lugdunensis*, *S. capitis*, *S. caprae*, *S. saprophyticus*, and *S. xylosus*. #*Streptococcus*species: *S. pyogenes*, *S. agalactiae*, *S. anginosus*, *S. bovis*, *S. constellatus*, *S. cristatus*, *S. gordonii*, *S. intermedius*, *S. milleri*, *S. mitis*, *S. mutans*, *S. oralis*, *S. parasanguinis*, *S. salivarius*, *S. sanguinis*, *S. thermophilus*, *S. vestibularis*, and *Viridans streptococci*). (DOCX 16 kb)

Additional file 2: **Table S2.** Baseline patient characteristics. Abbreviations: NYHA: “New York Heart Association”; LVEF: left ventricular ejection fraction; APACHE II: “Acute Physiologic Assessment and Chronic Health Evaluation II”; ICU = intensive care unit. (DOCX 17 kb)

Additional file 3: **Table S3.** Microorganisms detected by SF and BC in the Intervention group and Control group. ^#^Not included in SF detection list. ^##^A group of *Staphylococcus* species (*S. epidermidis*, *S. haemolyticus*, *S. hominis*, *S. pasteuri*, *S. warneri*, *S. cohnii*, *S. lugdunensis*, *S. capitis*, *S. caprae*, *S. saprophyticus*, and *S. xylosus* including *S. epidermidis*, *S. haemolyticus*, *S. hominis*, *S. pasteuri*, *S. warneri*, *S. cohnii*, *S. lugdunensis*, *S. capitis*, *S. caprae*, *S. saprophyticus*, and *S. xylosus). (DOCX 16 kb)*

Additional file 4: **Table S4.** Antimicrobial consumption in DOT/1000 patients-day according to the prescribed antimicrobial during the study. Abbreviations: DOT/1000PD: days of therapy/1000 patients-day; IQR: interquartile range; #: Other G neg antimicrobial: piperacillin-tazobactam, fluoroquinolones, cephalosporins, aminoglycosides, trimethoprim-sulfamethoxazol (DOCX 15 kb)

Additional file 5: **Figure S1.** Design of the study. (TIFF 778 kb)

Additional file 6: **Figure S2.** Protocol for the empirical antimicrobial therapy. (TIFF 627 kb)

Additional file 7: **Figure S3.** Flow chart. (TIFF 338 kb)

Additional file 8: Microbiological procedures. (DOCX 19 kb)

## Abbreviations

BC: Blood cultures
DOT: Days of therapy
PD: Patient day
SD: Standard deviation
SF: Septi*Fast*
