# Supplementary material for: The effect of a rapid molecular blood test on the use of antibiotics for nosocomial sepsis: a randomized clinical trial
Source: J Intensive Care. 2019 Jul 22;7:37. doi: 10.1186/s40560-019-0391-3 (PMC6647273; doi:10.1186/s40560-019-0391-3)
Supplement: Supplementary file 1 — Table S1. Microorganisms detected by the LightCycler® SeptiFast assay. Abbreviations: CoNS: coagulase-negative Staphylococcusspecies (S. epidermidis, S. haemolyticus, S. hominis, S. pasteuri, S. warneri, S. cohnii, S. lugdunensis, S. capitis, S. caprae, S. saprophyticus, and S. xylosus. #Streptococcusspecies: S. pyogenes, S. agalactiae, S. anginosus, S. bovis, S. constellatus, S. cristatus, S. gordonii, S. intermedius, S. milleri, S. mitis, S. mutans, S. oralis, S. parasanguinis, S. salivarius, S. sanguinis, S. thermophilus, S. vestibularis, and Viridans streptococci). (DOCX 16 kb) [file 40560_2019_391_MOESM1_ESM.docx]

Additional file 1: Table S1. Microorganisms detected by the LightCycler® Septi*Fast* assay.

| Gram-Negative Bacteria | Gram-Positive Bacteria | Fungus |
| --- | --- | --- |
| *Escherichia coli* | *Staphylococcus aureus* | *Candida albicans* |
| *Klebsiella pneumoniae/oxytoca* | *CoNS* | *Candida tropicalis* |
| *Serratia marcescens* | *Streptococcus pneumoniae* | *Candida parapsilosis* |
| *Enterobacter cloacae/aerogenes* | *Streptococcus spp#* | *Candida krusei* |
| *Proteus mirabilis* | *Enterococcus faecium* | *Candida glabrata* |
| *Pseudomonas aeruginosa* | *Enterococcus faecalis* | *Aspergillusfumigatus* |
| *Acinetobacter baumannii* |  |  |
| *Stenotrophomonas maltophilia* |  |  |

Abbreviations: CoNS: coagulase-negative *Staphylococcus* species (*S. epidermidis*, *S. haemolyticus*, *S. hominis*, *S. pasteuri*, *S. warneri*, *S. cohnii*, *S. lugdunensis*, *S. capitis*, *S. caprae*, *S. saprophyticus*, and *S. xylosus*. #*Streptococcus* species: *S. pyogenes*, *S. agalactiae*, *S. anginosus*, *S. bovis*, *S. constellatus*, *S. cristatus*, *S. gordonii*, *S. intermedius*, *S. milleri*, *S. mitis*, *S. mutans*, *S. oralis*, *S. parasanguinis*, *S. salivarius*, *S. sanguinis*, *S. thermophilus*, *S. vestibularis*, and *Viridans streptococci)*.
