# Supplementary material for: The effect of a rapid molecular blood test on the use of antibiotics for nosocomial sepsis: a randomized clinical trial
Source: J Intensive Care. 2019 Jul 22;7:37. doi: 10.1186/s40560-019-0391-3 (PMC6647273; doi:10.1186/s40560-019-0391-3)
Supplement: Supplementary file 2 — Table S2. Baseline patient characteristics. Abbreviations: NYHA: “New York Heart Association”; LVEF: left ventricular ejection fraction; APACHE II: “Acute Physiologic Assessment and Chronic Health Evaluation II”; ICU = intensive care unit. (DOCX 17 kb) [file 40560_2019_391_MOESM2_ESM.docx]

Additional file 2: Table S2.Baseline patient characteristics.

| Variable | Intervention Group  (n=100) | Control Group  (n=100) |
| --- | --- | --- |
| Age (years), median (IQR) | 65 (57 - 75) | 64 (56 - 72) |
| Gender, male | 71 (71.0%) | 66 (66.0%) |
| Pre-existing conditions |  |  |
| Diabetes mellitus | 35 (35.0%) | 39 (39.0%) |
| Chronic kidney disease | 61 (61.0%) | 61 (61.0%) |
| Renal replacement therapy | 4 (4.0%) | 7 (7.0%) |
| Creatinine clearance < 30 mL/min/1.73 m^2^ | 37 (37.0%) | 44 (44.0%) |
| Heart failure | 53 (53.0%) | 63 (63.0%) |
| Heart failure, NHYA classification |  |  |
| I | 4 (7.5%) | 13 (20.6%) |
| II | 7 (13.2%) | 10 (15.9%) |
| III | 29 (54.7%) | 29 (46.0%) |
| IV | 13 (24.5%) | 11 (17.5%) |
| Heart failure, NHYA classification≥3 | 42 (42.0%) | 40 (40.0%) |
| LVEF (%), median (IQR) | 40 (25 - 57) | 40 (25 - 55) |
| LVEF ≤ 35% | 49 (49.0%) | 45 (45.0%) |
| Causes of hospital admission, n (%) |  |  |
| Heart failure | 31 (31.0%) | 43 (43.0%) |
| Myocardial infarction | 29 (29.0%) | 21 (21.0%) |
| Cardiac arrhythmia | 9 (9.0%) | 18 (18.0%) |
| Infection | 9 (9.0%) | 6 (6.0%) |
| Coronary insufficiency | 8 (8.0%) | 3 (3.0%) |
| Others | 14 (14.0%) | 7 (7.0%) |
| Valvular heart disease | 0 (0%) | 2 (2.0%) |
| APACHE II score, median (IQR) | 15 (12 - 19) | 15 (10 - 19) |
| Intraaortic balloon pump at randomization | 25 (25.0%) | 18 (18.0%) |
| Mechanical ventilation at randomization | 49 (49.0%) | 46 (46.0%) |
| ICU, n (%) | 89 (89.0%) | 90 (90.0%) |

Abbreviations: NYHA: “New York Heart Association”; LVEF: left ventricular ejection fraction; APACHE II: “Acute Physiologic Assessment and Chronic Health Evaluation II”; ICU = intensive care unit.
