# Supplementary material for: The effect of a rapid molecular blood test on the use of antibiotics for nosocomial sepsis: a randomized clinical trial
Source: J Intensive Care. 2019 Jul 22;7:37. doi: 10.1186/s40560-019-0391-3 (PMC6647273; doi:10.1186/s40560-019-0391-3)
Supplement: Supplementary file 3 — Table S3. Microorganisms detected by SF and BC in the Intervention group and Control group. #Not included in SF detection list. ##A group of Staphylococcus species (S. epidermidis, S. haemolyticus, S. hominis, S. pasteuri, S. warneri, S. cohnii, S. lugdunensis, S. capitis, S. caprae, S. saprophyticus, and S. xylosus including S. epidermidis, S. haemolyticus, S. hominis, S. pasteuri, S. warneri, S. cohnii, S. lugdunensis, S. capitis, S. caprae, S. saprophyticus, and S. xylosus). (DOCX 16 kb) [file 40560_2019_391_MOESM3_ESM.docx]

Additional file 3: Table S3. Microorganisms detected by SF and BC in the Intervention group and Control group.

| Microorganism | Intervention Group  Positive SF  (n=19) | Control Group  Positive BC  (n=25) |
| --- | --- | --- |
| Gram-negative |  |  |
| *Enterobacter cloacae/aerogenes* | 4 | 2 |
| *Escherichia coli* | 2 | 1 |
| *Klebsiella pneumoniae/oxytoca* | 4 | 7 |
| *Serratia marcescens* | 1 | 0 |
| *Acinetobacterbaumanii* | 1 | 2 |
| *Pseudomonas aeruginosa* | 4 | 1 |
| *Stenotrophomonas maltophilia* | 0 | 2 |
| *Morganella morganii#* | - | 1 |
| Gram-positive |  |  |
| *Staphylococcus aureus* | 4 | 8 |
| *Coagulase negative staphylococci^##^* | 1 | 1 |
| *Rothiaspp^#^* | - | 1 |
| Number of microorganisms | 21 | 26 |
| Number of patients | 19 | 25 |

^#^Not included in SF detection list; ^##^A group of *Staphylococcus* species (*S. epidermidis*, *S. haemolyticus*, *S. hominis*, *S. pasteuri*, *S. warneri*, *S. cohnii*, *S. lugdunensis*, *S. capitis*, *S. caprae*, *S. saprophyticus*, and *S. xylosus* including *S. epidermidis*, *S. haemolyticus*, *S. hominis*, *S. pasteuri*, *S. warneri*, *S. cohnii*, *S. lugdunensis*, *S. capitis*, *S. caprae*, *S. saprophyticus*, and *S. xylosus).*
