# Supplementary material for: The effect of a rapid molecular blood test on the use of antibiotics for nosocomial sepsis: a randomized clinical trial
Source: J Intensive Care. 2019 Jul 22;7:37. doi: 10.1186/s40560-019-0391-3 (PMC6647273; doi:10.1186/s40560-019-0391-3)
Supplement: Supplementary file 4 — Table S4. Antimicrobial consumption in DOT/1000 patients-day according to the prescribed antimicrobial during the study. Abbreviations: DOT/1000PD: days of therapy/1000 patients-day; IQR: interquartile range; #: Other G neg antimicrobial: piperacillin-tazobactam, fluoroquinolones, cephalosporins, aminoglycosides, trimethoprim-sulfamethoxazol (DOCX 15 kb) [file 40560_2019_391_MOESM4_ESM.docx]

Additional file 4: Table S4. Antimicrobial consumption in DOT/1000 patients-day according to the prescribed antimicrobial during the study.

| Antimicrobial consumption: | **All patients** | | | **Positive test** | | |
| --- | --- | --- | --- | --- | --- | --- |
|  | Intervention Group | Control Group | P value | Intervention Group | Control Group | P value |
| DOT/1000 PD, median, (IQR) | (n=100) | (n=100) |  | (n=19) | (n=25) |  |
| Glycopeptides, linezolid, daptomycin, and oxacillin | 786 (554-1000) | 866 (641 - 1000) | 0.259* | 71 (71-1000) | 786 (354-1000) | 0.013* |
| MEROPENEM | 393 (0 - 706) | 352 (0 - 623) | 0.825* | 179 (0 - 482) | 250 (0 - 518) | 0.725* |
| POLYMIXIN | 0 (0 - 386) | 0 (0 - 387) | 0.703* | 18 (0 - 473) | 0 (0 - 402) | 0.735* |
| OTHER G neg ANTIMICROBIAL | 0 (0 - 386) | 107 (0 - 525) | 0.253* | 33 (0 - 321) | 211 (43 - 1000) | 0.045* |

*Mann-Whitney test

Abbreviations: DOT/1000PD: days of therapy/1000 patients-day; IQR: interquartile range; #: Other G neg antimicrobial: piperacillin-tazobactam, fluoroquinolones, cephalosporins, aminoglycosides, trimethoprim-sulfamethoxazol
