# Supplementary figures and images for: The effect of a rapid molecular blood test on the use of antibiotics for nosocomial sepsis: a randomized clinical trial
Source: J Intensive Care. 2019 Jul 22;7:37. doi: 10.1186/s40560-019-0391-3 (PMC6647273; doi:10.1186/s40560-019-0391-3)

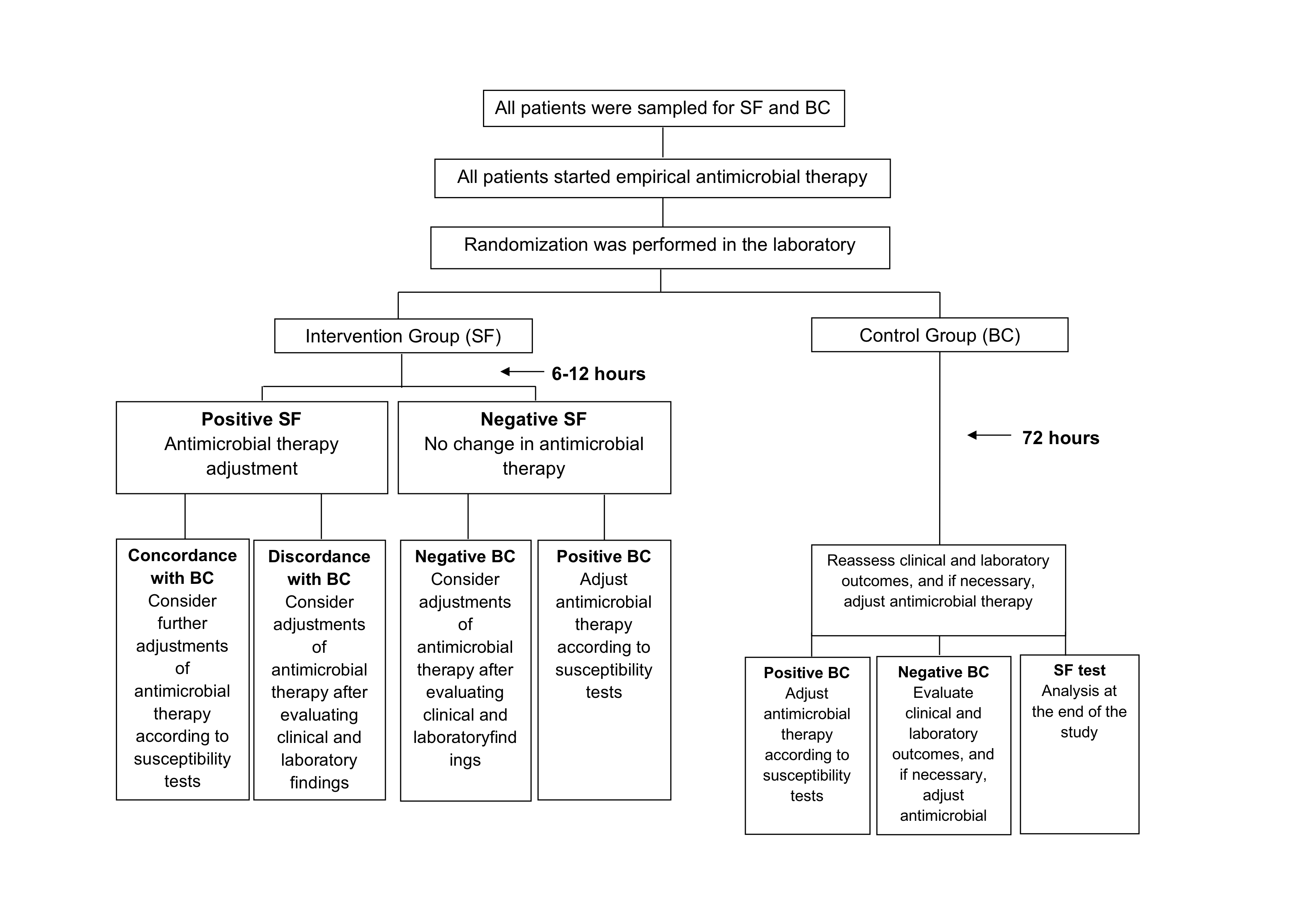

Supplement: Supplementary file 5 — Figure S1. Design of the study. (TIFF 778 kb) [file 40560_2019_391_MOESM5_ESM.tiff]

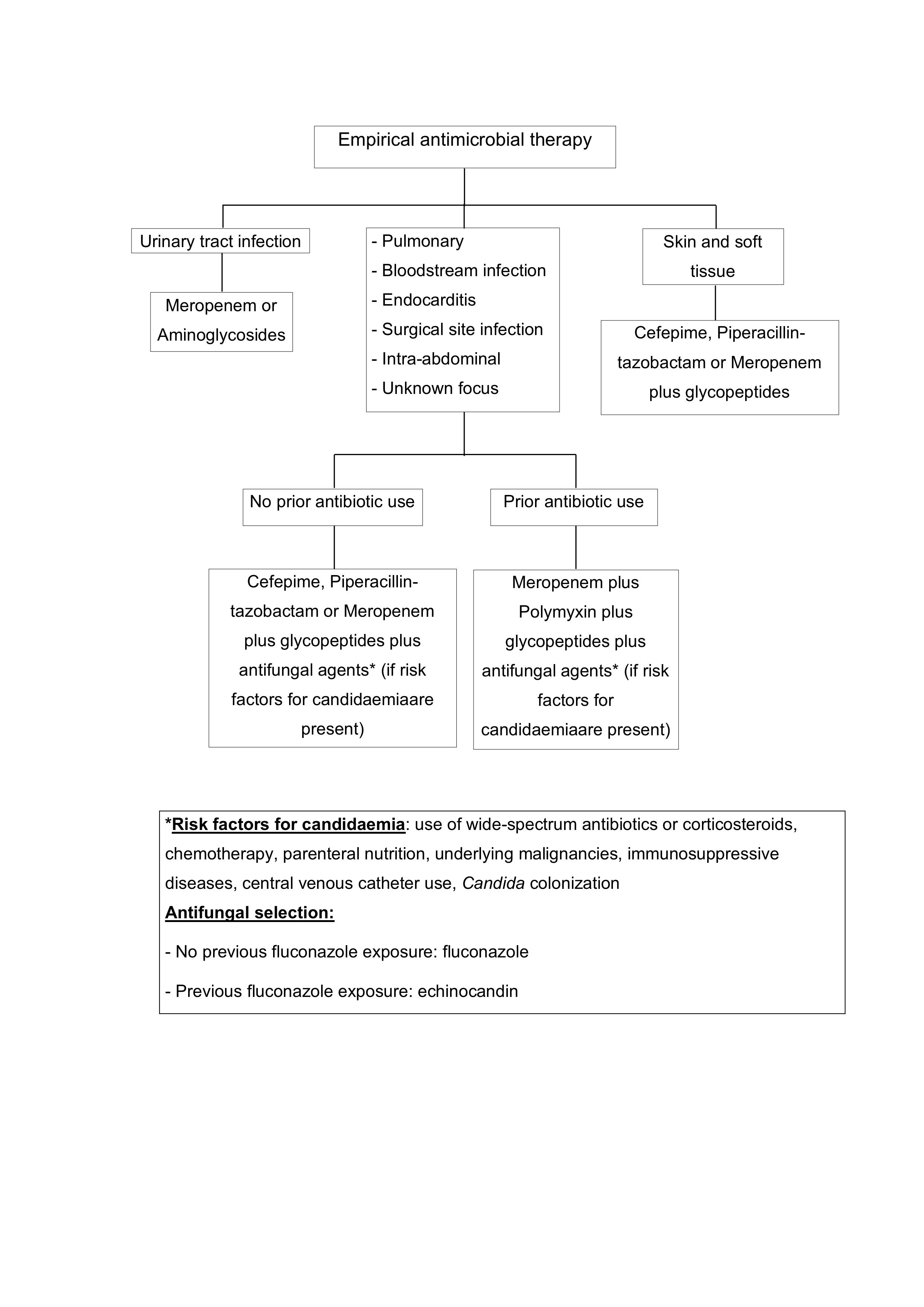

Supplement: Supplementary file 6 — Figure S2. Protocol for the empirical antimicrobial therapy. (TIFF 627 kb) [file 40560_2019_391_MOESM6_ESM.tiff]

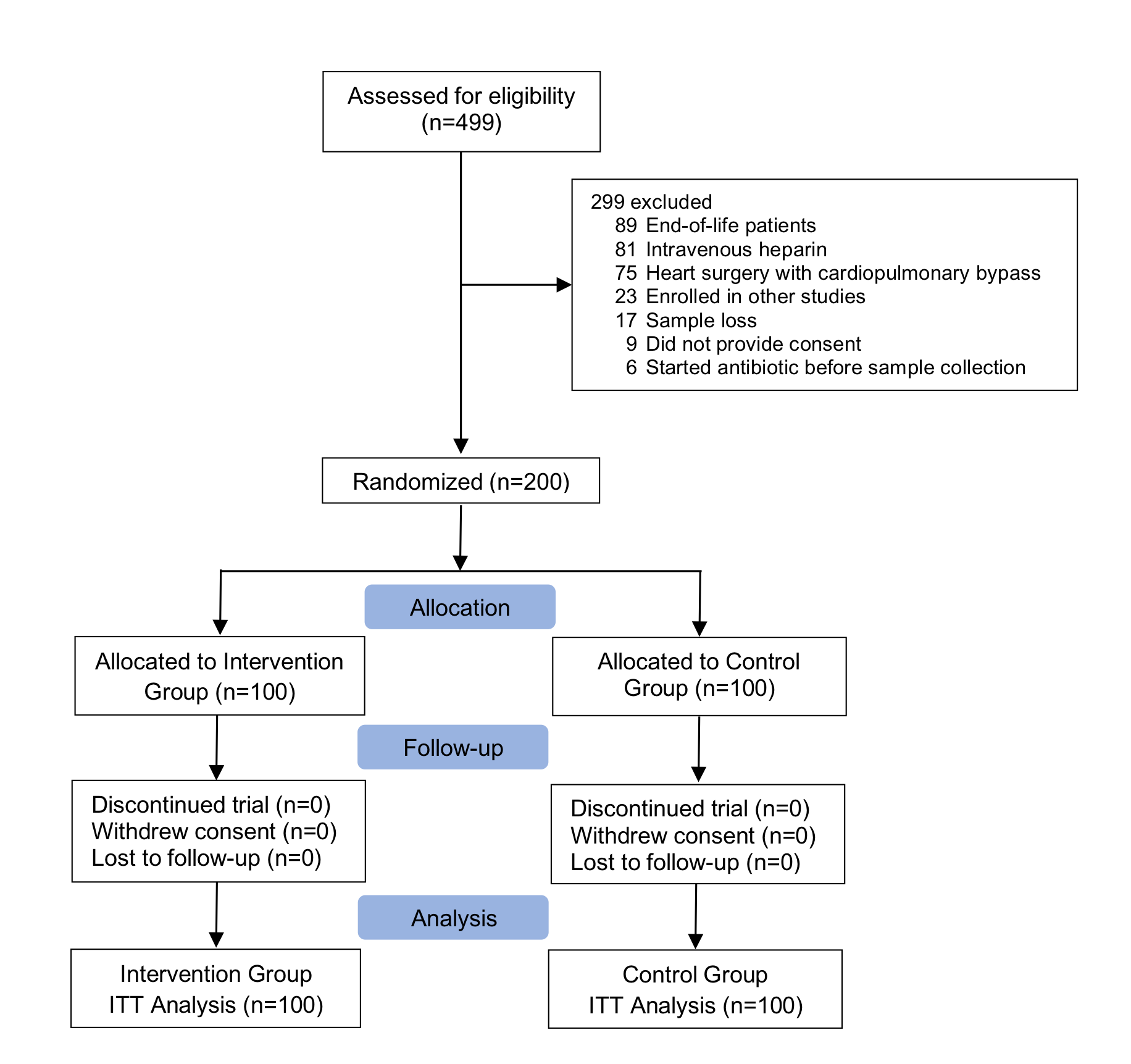

Supplement: Supplementary file 7 — Figure S3. Flow chart. (TIFF 338 kb) [file 40560_2019_391_MOESM7_ESM.tiff]
