# Supplementary material for: The effect of a rapid molecular blood test on the use of antibiotics for nosocomial sepsis: a randomized clinical trial
Source: J Intensive Care. 2019 Jul 22;7:37. doi: 10.1186/s40560-019-0391-3 (PMC6647273; doi:10.1186/s40560-019-0391-3)
Supplement: Supplementary file 8 — Microbiological procedures. (DOCX 19 kb) [file 40560_2019_391_MOESM8_ESM.docx]

Additional file 8

**Microbiological procedures**

Blood samples were aseptically collected by venipuncture according to standardized procedures. Two sets of aerobic and anaerobic cultures were obtained (8-10 mL per bottle) with 4 mL of blood into K2EDTA tubes (Vacutainer®, Becton Dickison, UK) for the SF assay.

The BC were processed in routine microbiology laboratory using the BACTEC FX (Becton Dickinson, USA) system for aerobes, anaerobes and fungi. Blood cultures for aerobes and anaerobes were incubated for 5 days and 42 days for fungi. When BC was positive, microorganisms were identified by MALDI-TOF (matrix-assisted laser desorption/ionization time-of-flight mass spectrometry) and susceptibility testing by disk diffusion test and or MIC (minimum inhibitory concentration)detection accordingly to the laboratory protocol. Gram-stain results were reported in the electronic medical record without extra notification to the care team. The final microorganism identification and susceptibility test were completed until 96 hours and reported in the electronic medical record available to all care team.

The SF assay was designed to detect microbial DNA of 25 microorganisms in whole blood samples (Table 1). The test was done in molecular laboratory according to manufacturer’s instruction. To avoid DNA contamination the specimen was prepared in a laminar flow box and the PCR reaction mix in an UltraViolet box. Mechanical lysis of specimens was performed using the SeptiFast Lys Kit M Grade. A negative control was used in each run, and an internal control was added to each sample before DNA purification. DNA amplification was processed on the LightCycler 2.0 instrument in three different PCR reactions: gram-positive bacteria, gram-negative bacteria, fungi using the internal transcribed spacer region (ITS) located between 16S – 23S ribosomal DNA sequences of bacteria and 18S-5.8S ribosomal DNA sequences of fungi as targets. The identification of species was made by melting curve analysis using specifically designed LightCycler® SeptiFast Identification Software. [[13](#_ENREF_12)]
